# Research Advances in Neuroblast Migration in Traumatic Brain Injury

**DOI:** 10.1007/s12035-024-04117-4

**Published:** 2024-03-20

**Authors:** Na Wu, Wenlang Li, Qiang Chen, Meng Chen, Siyuan Chen, Chongjie Cheng, Yimin Xie

**Affiliations:** 1 Department of Pediatric Surgery, Chongqing University Three Gorges Hospital, Wanzhou District, No. 165 Xincheng Road, Wanzhou District, Chongqing, 404100 China; 2 Department of Neurosurgery, The First Affiliated Hospital of Chongqing Medical University, Yuzhong District, Chongqing, China; 3 Department of Anesthesiology, The Second Affiliated Hospital of Chongqing Medical University, Yuzhong District, Chongqing, China

**Keywords:** Neuroblast migration, Traumatic brain injury, Neurogenesis, Neuronal migration, Vascular migration

## Abstract

Neuroblasts were first derived from the adult mammalian brains in the 1990s by Reynolds et al. Since then, persistent neurogenesis in the subgranular zone (SGZ) of the hippocampus and subventricular zone (SVZ) has gradually been recognized. To date, reviews on neuroblast migration have largely investigated glial cells and molecular signaling mechanisms, while the relationship between vasculature and cell migration remains a mystery. Thus, this paper underlines the partial biological features of neuroblast migration and unravels the significance and mechanisms of the vasculature in the process to further clarify theoretically the neural repair mechanism after brain injury. Neuroblast migration presents three modes according to the characteristics of cells that act as scaffolds during the migration process: gliophilic migration, neurophilic migration, and vasophilic migration. Many signaling molecules, including brain-derived neurotrophic factor (BDNF), stromal cell-derived factor 1 (SDF-1), vascular endothelial growth factor (VEGF), and angiopoietin-1 (Ang-1), affect vasophilic migration, synergistically regulating the migration of neuroblasts to target areas along blood vessels. However, the precise role of blood vessels in the migration of neuroblasts needs to be further explored. The in-depth study of neuroblast migration will most probably provide theoretical basis and breakthrough for the clinical treatment of brain injury diseases.

**Graphical Abstract:**

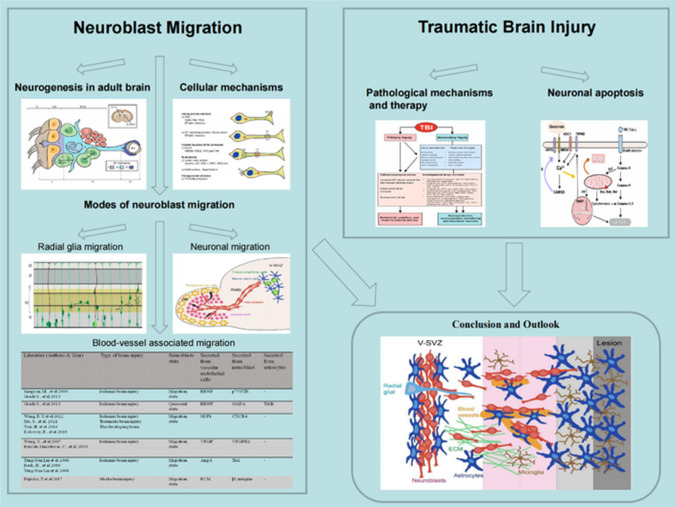

## Introduction

Traumatic brain injury (TBI) has high mortality and morbidity, leading to severe neurological dysfunction [1, 2]. Neurogenesis involving the maintenance and self-renewal of neural stem cells (NSCs), as well as the survival, migration, maturation, and integration of neuroblasts [1], has underlined therapeutic options for treating TBI. Neuroblasts are immature cells of neuronal lineage that migrate to target brain regions from their birthplaces to become neurons and integrate into neural networks [3]. Neuroblasts were first derived from the adult mammalian brains in the 1990s by Reynolds et al. [4]. Since then, persistent neurogenesis in the subgranular zone (SGZ) of the hippocampus and subventricular zone (SVZ) has gradually been recognized [5–12]. Endogenous neurogenesis following brain injury occurs as follows [13–15]: neuroblasts in the SVZ migrate toward the damaged tissue and contribute to neuronal repair, which could be a lengthy process with multiple influencing factors [16–20].

Two distinct modes of neuroblast migration have been well recognized so far based on the direction of cell migration [18]: radial and tangential migration. However, neuroblast migration can be classified into neuronal migration, glial cell migration, and vascular migration, according to the medium of cell migration. To date, reviews on neuroblast migration have largely investigated glial cells and molecular signaling mechanisms, while the relationship between vasculature and cell migration remains a mystery. Thus, this paper underlines the partial biological features of neuroblast migration and unravels the significance and mechanisms of the vasculature in the process to further clarify theoretically the neural repair mechanism after brain injury and provide reference for clinical treatment of this diseased condition.

## Traumatic Brain Injury

### Pathological Mechanisms and Therapy of Traumatic Brain Injury (Fig. 1)

**Fig. 1:**
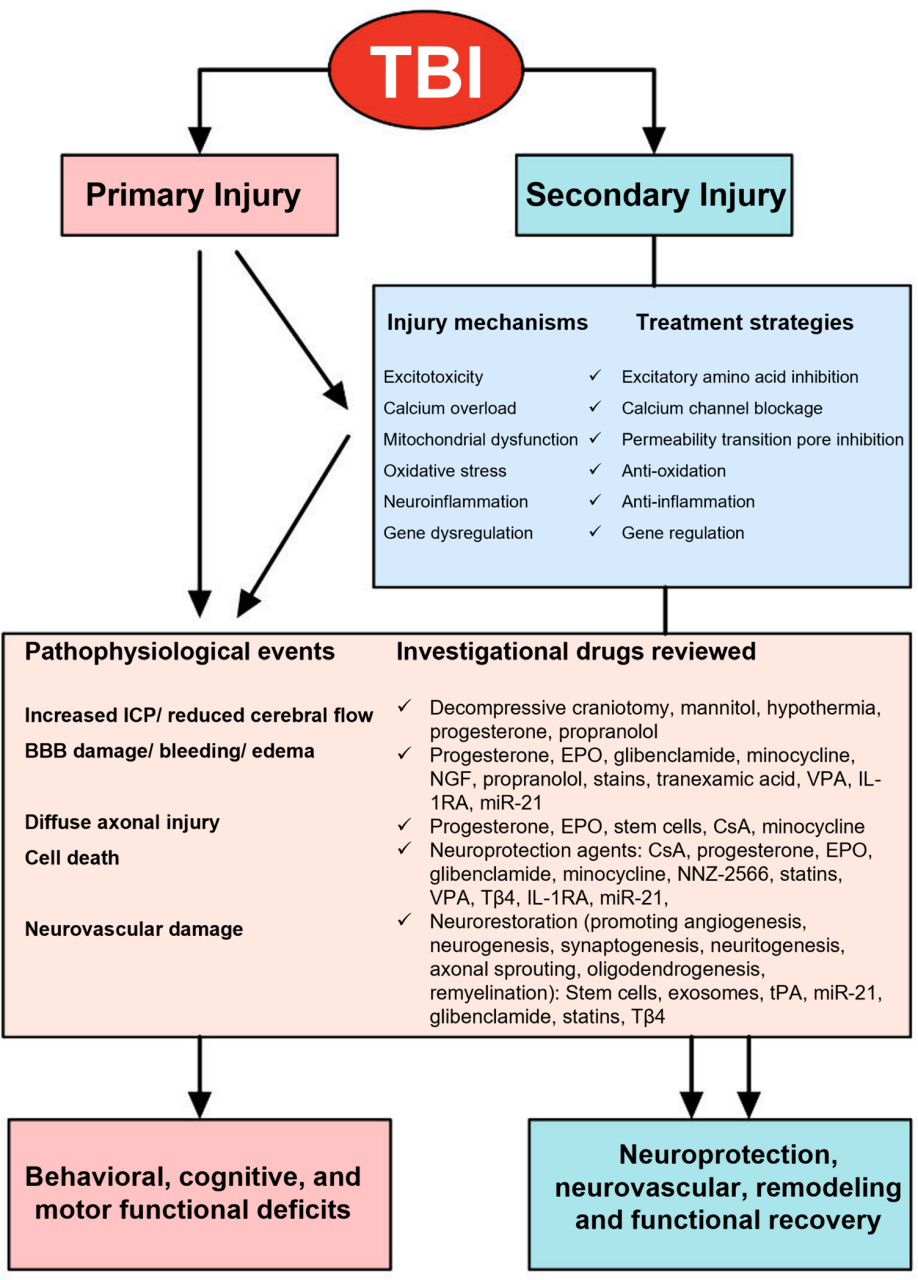
Simplified overview of pathophysiology and therapy of TBI [21]. TBI, traumatic brain injury; ICP, intracranial pressure; BBB, blood–brain barrier; EPO, erythropoietin; NGF, nerve growth factor; VPA, valproic acid; IL-1RA, interleutin-1receptor antagonist; miR-21, microRNA-21; CsA, cyclosporine A; NNZ-2566, synthetic analogue of the endogenous N-terminus tripeptide glycine-proline-glutamate; Tβ4, thymosin beta 4; tPA, tissue plasminogen activator

TBI can result from exposure to a blow or blast, rapid head deceleration or acceleration, and skull penetration, causing not a single pathophysiological event at the time of injury but a complex continuous disease process [21, 22]. After TBI, structural damage and functional deficits occur due to both primary and secondary injury mechanisms [2, 21]. Primary injury of mechanical tissue deformation and injury not only leads to cell death, shearing, and tearing of blood vessels, neuron, glia, and axon, but also initiates secondary injury cascades, such as excitotoxicity and oxidative stress. Excitotoxicity is nonspecific depolarization and release of excitatory neurotransmitters, glutamate, and aspartate, which bind to glutamate receptors and induce massive influx of calcium called as calcium overload. Calcium overload activates calcium-dependent phospholipases, proteases, and endonucleases, damaging cell membrane, cytoskeleton, and nucleic acids, respectively. Mitochondria sequester intracellular calcium may cause mitochondrial permeability pore opening, energy deficits, free radical formation, and initiation of apoptosis. Also, TBI initiates oxidative stress because of significantly increased formation of oxygen and nitrogen reactive species, which oxidize lipids, proteins, and nuclei acids. Furthermore, TBI upregulates transcription factors, inflammatory mediators, and neuroprotective genes but downregulates neurotransmitter receptors and neurotransmitter release mechanisms. Increased expression of detrimental cytokines and chemokines induces brain edema, blood–brain barrier damage, and apoptosis. These complex cascades subsequently induce blood–brain barrier damage, hemorrhage, edema, increased ICP, altered cerebral flow, ischemia/hypoxia, metabolic deficits, apoptosis, diffuse axonal injury, demyelination, progressive atrophy of both grey and white matter, which collectively cause cell death, brain neurodegeneration, and functional deficits. However, accumulative experimental and clinical data over the past decade have indicated that the adult brain is capable of, limited though, structural and functional reorganization after injury, possibly, contributing to spontaneous functional recovery. Recent new interventions targeting multiple secondary injury mechanisms and promoting neuroplasticity mechanisms have improved functional recovery in animal models of TBI.

Effective therapeutic strategies for TBI are lacking due to its heterogeneous nature [2, 21, 22]. Two strategic approaches have been developed: neuroprotective treatment and neurorestorative treatment [21]. Neuroprotective treatment targets the injured brain to reduce/prevent secondary injury and neural cell death, as well as reduce the lesion size; neurorestorative treatment improves neurological recovery by treating the entire central nervous system (CNS) to promote neurovascular remodeling including angiogenesis, neurogenesis, oligodendrogenesis, and dendrite/axon outgrowth [1, 2, 21]. However, ideal treatment for TBI remains to be further explored due to its multiple complications during pathogenesis.

### Neuronal Apoptosis After Traumatic Brain Injury (Fig. 2)

**Fig. 2:**
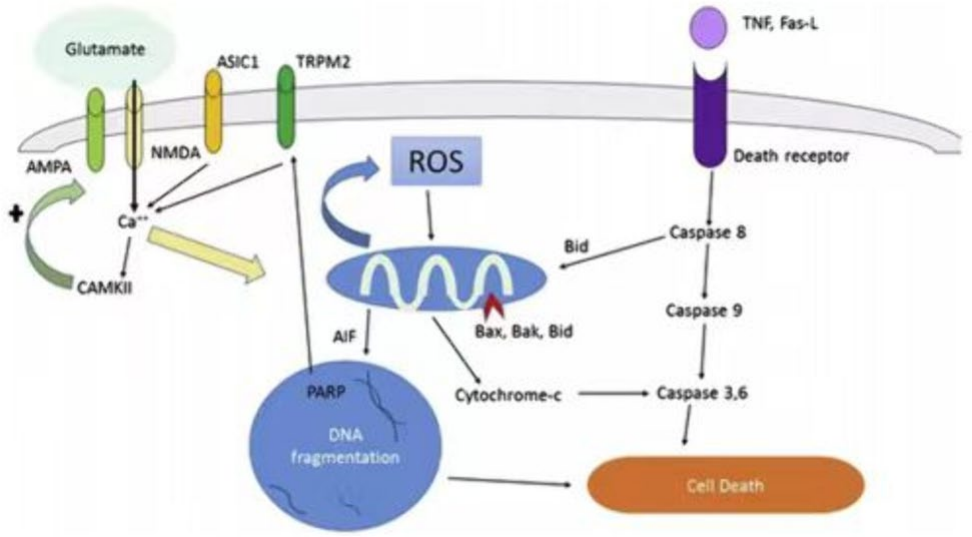
Glutamate, reactive oxygen species, and apoptotic cell death pathways [22]. Fas-L, Fas ligand; ROS, reactive oxygen species

Neuronal apoptosis is a genetically controlled mechanism of cell death involved in the regulation of tissue homeostasis. Biochemical events lead to characteristic cell changes (morphology) and cell death [22]. These morphological changes include cell blebbing, cell shrinkage, nuclear fragmentation, chromatin condensation, chromosomal DNA fragmentation, and messenger RNA decay. Triggers of apoptosis include oxygen free radicals, death receptor ligation, DNA damage, protease activation, and ionic imbalance. Both extrinsic (Fas and other tumor necrosis factor receptor superfamily members and ligands) and intrinsic (mitochondria-associated) pathways involved in apoptosis are found in the cytoplasm. The extrinsic pathway is triggered by death receptor engagement, initiating a signaling cascade mediated by caspase-8 activation, whereas the intrinsic pathway is engaged when various apoptotic stimuli trigger the release of cytochrome c from mitochondria independently of caspase-8 activation. Both pathways ultimately cause caspase-3 activation, degrading cellular proteins necessary to maintain cell survival and integrity. Besides, there is a complex interplay of the Bcl-2 family of proteins, which either promote (Bax, Bak, Bad, Bim, Bid) or prevent (Bcl-2, Bcl-xL, Bcl-w) injury. Bcl-2 and its family member, Bcl-xL, are among the most powerful death-suppressing proteins which inhibit both caspase-dependent and caspase-independent cell death. Apoptosis-inducing factor (AIF), a caspase-independent apoptotic pathway, is stored within the same mitochondrial compartment as cytochrome c. DNA damage via PARP activation and oxidative or excitotoxic stress release AIF, which is translocated to the nucleus to induce apoptosis. Figure 2 shows these pathways.

## Neurogenesis in Adult Brain

**Fig. 3:**
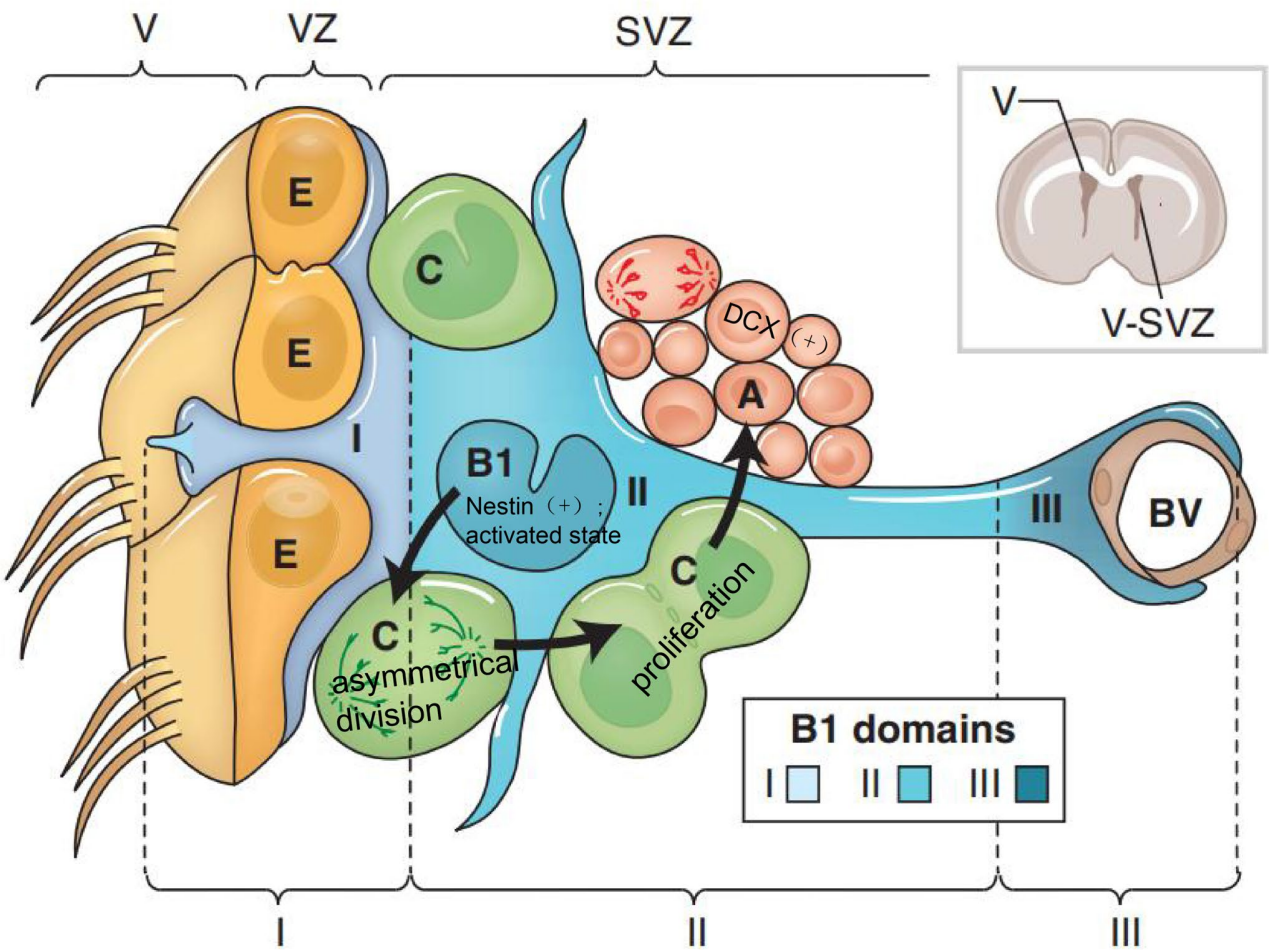
Cellular composition of the ventricular–subventricular zone (V-SVZ) [24]. Coronal section of adult mouse brain is shown in the upper right. The V-SVZ region indicated by the black arrow is shown enlarged in the lower left. Type B1 cells (blue; GFAP-positive) are the astrocytes that serve as the V-SVZ stem cell. These can divide and produce type C cells (green; Nestin-positive), which are rapidly dividing, transit amplifying cells. Type C cells give rise to type A cells (red; DCX-positive), the migratory neuroblasts. A blood vessel (BV, brown) is shown at the right. The apical surface of type B1 cells has a primary cilium and makes contact with the ventricle, which is at the left. These apical surfaces are found at the center of a “pinwheel” composed of multiciliated ependymal cells (yellow). The V-SVZ can be subdivided into three domains based on the structure and spatial arrangement of type B1 cells: Domain I (apical) contains the type B1 cells apical process and the body of ependymal cells; domain II (intermediate) contains the cell body of most type B1 cells, which are in contact with the type C and A cells; and domain III (basal) contains the B1 cell’s basal process with end-feet on blood vessels

Neurogenesis involves the maintenance and self-renewal of neural stem cells (NSCs), as well as the survival, migration, maturation, and integration of neuroblasts. The SGZ of the hippocampus and the SVZ are regions where adult neurogenesis mostly occurs [23, 24], the latter of which mainly contains the following architectures (Fig. 3): ependymal cells; type B1 cells, i.e., SVZ stem cells; type C cells, i.e., rapidly proliferating neuroblasts; and type A cells, i.e., migratory neuroblasts. Type B1 cells can exist in the quiescent state, with most of the cells being GFAP-positive, or in an activated state, being Nestin-positive. When activated, type B1 cells undergo asymmetrical division and turn into type C cells, which rapidly proliferates into DCX-positive, migratory type A cells [25]. Under normal circumstances, type A neuroblasts in the rostral migratory stream (RMS) migrate to the olfactory bulb (OB); in the event of brain injury, part of the neuroblasts move out of the RMS and migrate toward the focal area [23, 26].

## Cellular Mechanisms of Neuroblast Migration

Neuroblast migration, although diverse in modes and pathways, undergo three cytological events [27–29]: extension of the leading process, which has a growth cone at its distal tip to explore microenvironment; forward movement of the centrosome, and translocation of the nucleus, i.e., “nucleokinesis”; trailing process retraction. Recurrence of the above three events contributes to the overall movement of the neuron.

**Fig. 4:**
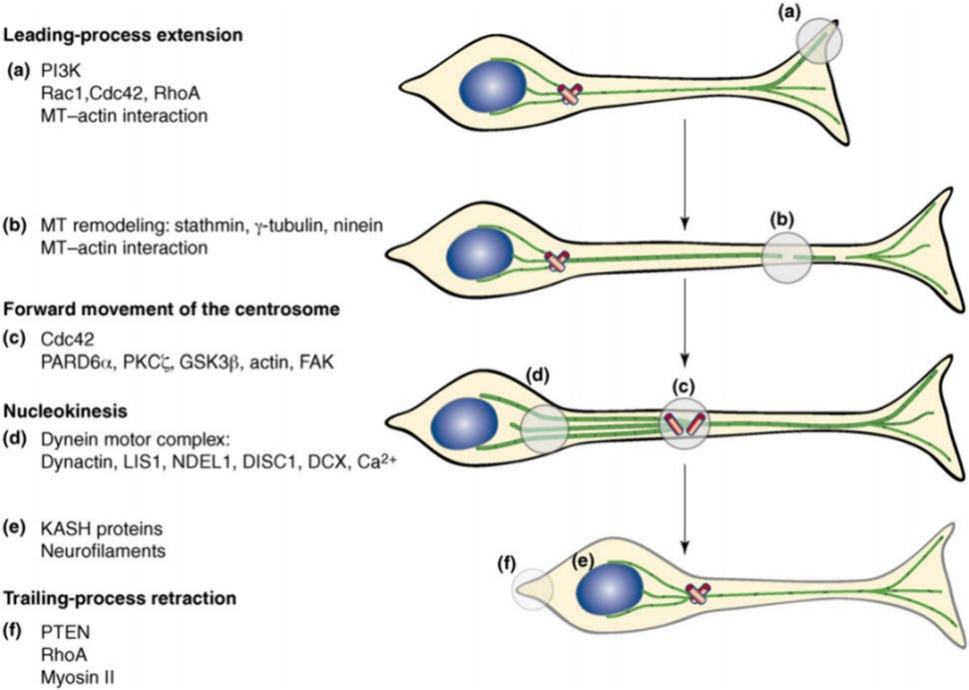
Steps in neuronal migration and molecules involved [19]. **a**, **b** Polarized extension of the leading process. **a** PI3K signaling at the front of the cell regulates the balance of activation of the Rho GTPases Cdc42, Rac1, and RhoA. Inhibition of RhoA enhances leading-process outgrowth, whereas inhibition of Rac1 and Cdc42 impairs neurite outgrowth. Microtubule plus ends are recruited to the cortical actin meshwork. **b** In the intermediate segment of the leading process, microtubules (MT, green) are loosely organized, probably owing to the destabilizing activity of stathmin. γ-Tubulin and the microtubule-related protein ninein show a wide distribution in migrating neurons. **c** Forward movement of the centrosome. Cdc42 is found mainly in the perinuclear region. Forward movement of the centrosome (red rods) involves PARD6α and its associated kinase PKCξ; reorientation of the centrosome requires the activity of GSK3β, PKCξ, and the actin cytoskeleton. Focal-adhesion kinase (FAK) also contributes to centrosomal dynamics. Both centrioles split during the advance of the soma. **d**, **e** Movement of the nucleus (blue oval) toward the centrosome (nucleokinesis).**d** Nucleokinesis requires a microtubule motor complex based on dynein; proteins interacting within this include dynactin, LIS1, NDEL1, DISC1, and DCX. DCX molecules are found attached to microtubules that extend from the centrosome to the perinuclear “cage.” Ca^2+^ signaling might also operate at this stage. **e** Various components of the KASH family of proteins anchor the nucleus to the centrosome and cell membrane. Neurofilaments might contribute to connecting the nucleus to the cell cortex. **f** Trailing-process retraction. PTEN signaling at the back regulates RhoA. Actomyosin contraction has a role in driving the nucleus toward the centrosome

Marin et al. [19] elaborated three events of neuroblast migration and their influencing molecules (Fig. 4). First, in the extension of the leading process, the PI3K signaling pathway plays a significant role, with RhoA, Rac1, and Cdc42 functioning as three critical regulatory molecules in this pathway. Inhibiting RhoA is thought to promote the growth of leading processes, while inhibiting Rac1 and Cdc42 can prevent its growth. At the tip of the leading process, the positive end of the microtubule binds to actin, forming a terminal web. While in the middle of the leading process, stathmin is a protein that takes the role of microtubule destabilizer. In addition, γ-tubulin, along with microtubule protein ninein, are vital for microtubule reconstruction, and exist extensively in the neurons [30]. Second, Cdc42 prevails primarily in the perinuclear region in the forward movement of the centrosome, which involves PARD6α and protein kinase PKCξ; while repositioning of centrioles involves GSK3β, PKCξ, and microfilaments. And again, the nucleus moves toward the centriole, and nuclear movement attributes to the participation of microtubule dynamic complex of dynein. Proteins interacting with the complex encompass dynactin, Ndel1, Lis1, DISC1, and DCX (doublecortin). DCX binds to the microtubules connecting the centriole to the nucleus, an event which may involve Ca2 + signaling [31, 32]. Different proteins of the KASH domain anchor the nucleus to the centriole and the cell membrane. The neurofilament may contribute to the binding of the nucleus to the cell cortex. Finally, the trailing process undergoes retraction, which is an event left to be explored in-depth, although PTEN signaling and actomyosin at the cell ends may play a great part.

## Modes of Neuroblast Migration

Two modes of neuroblast migration have been known based on the direction of migration: tangential and radial migration. The former one indicates that cells migrate in a direction parallel to the pial surface and travel to an appropriate site over a long distance, while the latter one refers to that cells follow a trajectory that is perpendicular to the neuroepithelial surface where the neurons migrate from inside out to a specific site to develop cortex. Regarding medium of migration, neuroblast migration is classified into gliophilic migration, neurophilic migration, and vasophilic migration [33–35].

### *Gliophilic* Migration *(Migration *Along Radial Glia Cell*)*

**Fig. 5:**
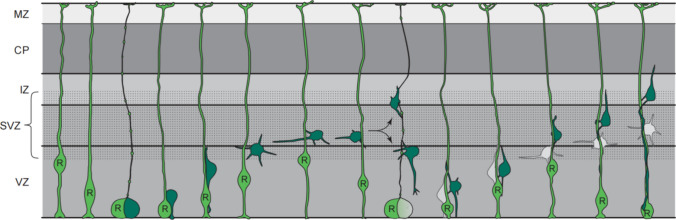
Glial-guided neuroblast migration [42]. Phase one involves radial movement of pyramidal neurons (dark green) from the site of origin at the ventricular surface to the subventricular zone (SVZ). In phase two, cells become multipolar and pause their migration in the lower intermediate zone (IZ) and subventricular zone (SVZ). Some neurons undergo phase three, which is characterized by retrograde motion toward the ventricle. Phase four is the final radial migration to the cortical plate (CP), guided by radial glial fibers. Radial glia (light green) remain mitotic, undergo interkinetic nuclear migration, and generate additional daughter cells (grey). MZ, marginal zone; R, radial glial cell; VZ, ventricular zone

Gliophilic migration is defined as migration of cells using long radial processes of radial glia as a scaffold [36–38]. Rakic et al. [39] proposed this term for the first time and described that in the development of neocortex, neuroblasts migrate along the radial processes vertically. Both in vivo [40, 41] and in vitro [42, 43] experiments showed that radial glial cells are major factors in the migration of neuroblasts. Neuroblasts migrate from the ventricular zone to the cortical plate in the following steps (Fig. 5): (1) Binding to radial glial cells; (2) Migrating as radial glial cells, presenting as reverse movement or lateral movement in the process of forward movement, so called dance sign [42]: neuroblasts do not appear to be tightly adhered to radial glial guide fibers at this stage, and are capable of moving tangentially. Some neuroblasts have been observed to move and return to their original locations. Some neuroblasts have been investigated to extend a process toward the ventricle. (3) Detaching from radial glial cells, and settling in the cortex, differentiating and finally maturing. Neuroblast migration requires complicated intermolecular interactions involving a variety of transmembrane receptors, intracellular signaling molecules, transcription factors (TF), extracellular matrix (ECM), diffusion factors, and adhesion factors [3, 6, 14, 17, 27, 41, 43–47]. Currently, “glial cell-derived signaling” and “complicated intermolecular interactions between long radial processes and neuroblasts” have attracted great attention.

Neuroblasts during migration can generate astrotactin, a glycoprotein that is the first factor shown to mediate neuron-glial interactions. In the cerebellar microcultures experiment, astrotactin involved in the adhesion of neuroblasts to neuron-glial cells increased [48], and mice without the expression of this factor showed decreased glial adhesion and radial migration. Integrin, which mediates intercellular and intercellular-matrix interactions, has also been ascertained to affect neuronal-glial cell adhesion in the radial migration. In mice brains without integrin expression, radial migration was significantly reduced [49] because function-blocking antibodies against integrin induced the detachment of migrating cells and radial glial cells. Real-time imaging reveals that NPCs stretch out small processes to wrap around the radial glial fibers along the migratory direction. As the neurons tightly bind to the glial cells, the cell body moves forward in a jumping manner. Radial glial cells guide neuronal migration, while neurons conversely affect function of radial glial cells. Neuron-glial cell adhesion and neuronal diffusing factors induce the extension of glial cell processes. For instance, migrating neurons in the cortex can generate glial growth factor (GGF), which promotes the maintenance and elongation of radial glial cells [50].

### Neurophilic Migration (Migration Along Neuron Chains)

**Fig. 6:**
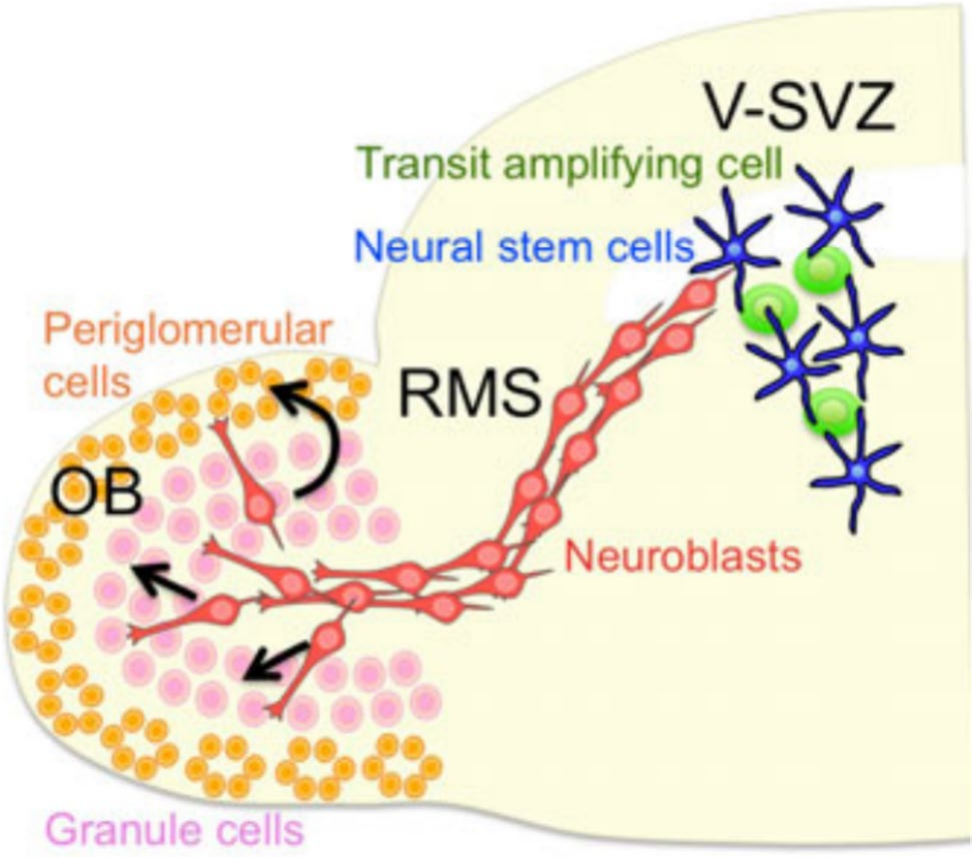
Migration of neuroblasts from V-SVZ to the olfactory bulb(OB) [17]. Illustration showing the migration of V-SVZ neuroblasts to the olfactory bulb. Neuroblasts (red) are generated in the V-SVZ by neural stem cells (blue) through intermediate progenitors called transit-amplifying cells (green). The neuroblasts form elongated, chain-like aggregates that migrate tangentially through the rostral migratory stream (RMS) toward the olfactory bulb. After reaching the olfactory bulb (OB) and detaching from the chain, individual neuroblasts migrate radially to the outer layer where they differentiate into olfactory interneurons, granule cells (pink), or periglomerular cells (orange) and are integrated into the olfactory neuronal circuitry

Neurophilic migration is distinct from glial cell migration in the way that migrating cells act as scaffolds for each other and influence migration of each other, as is represented by neuroblast migration from the SVZ to the OB. Meanwhile, upon reaching the OB, these cells will differentiate into interneurons. Numerous signaling molecules are related to this migration pathway. Chain migration is a unique manifestation of cell migration in the RMS, where neuroblasts migrate from the V-SVZ to the OB in a chain-like interlocking arrangement through the connections between them [17, 51] (Fig. 6). Signaling molecules involved in the migration chain of neuroblasts include PSA-NCAM (Polysialic acid-Neural cell adhesion molecule), slit family, integrin family, unknown ASTN-derived factor, cyc-lin-dependent kinase, ErbB4, GABA, and prokineticin 2 receptor (PKR2), some of which join in the connections between neuroblasts, and some control cell motility or cell-ECM interactions [52]. Further studies are needed to clarify the mechanisms of why and when neuroblasts choose such migration pattern.

### Vasophilic Migration (Migration Along Blood Vessels)

In vasophilic migration, cells undergoing migration use blood vessels as scaffolds [33]. Experiments show that cerebral neuroblasts in both normal physiological [53] and pathological states [54] may also migrate using blood vessels as scaffolds [33–35].

The experimental results of Ono et al. [55] firstly verified that the migration of neuroblasts depends on vascular guidance. The neuroblasts in the OB are closely connected with the vasculature. Once they reach the OB, the neuroblasts separate from the migratory chain and no longer migrate tangentially, initiating radial migration to different sites. Chen Wenjing et al. [56] corroborated blood vessels and radial glial cells remain consistent distribution and direction during cerebellar development and keep a mutual induction relationship, which suggest that blood vessels could guide the migration of neuroblasts at the same time.

Yu Qi et al. [57] demonstrated that cloned neuronal cell lines implanted in the mouse brain also migrate along the vasculature. In addition to OB, neuroblasts migration along the vasculature occur also in RMS. Blood vessels are densely packed in RMS, where the vessels are parallel to the migratory stream and in close proximity to the migrating cells, and the specialized substrates generated by the vessels in the SVZ stop at the ventricular cells, allowing the ventricles to maintain contact with the NSC-rich sites in the SVZ, hence maintaining their microenvironment [58]. Parallel vessels are clustered for reconstruction in the center of the OB where tangential migration is changed to radial migration correspondingly, and blood vessels are clustered and parallel to tangential migration (or radial migration). These findings showed that neuroblasts during migration take the blood vessels as a scaffold and migrate from the SVZ to the granule cell layer and the glomerular layer of the OB. Real-time imaging reveals two complementary modes of neuroblast migration. In the first mode, neuroblasts present linear migration, cell bodies, and anterior processes being close to the blood vessels; in the second mode, only the processes are close to the blood vessels. The probable reason for the second mode is the migrating NPCs encounter some physical constraints (e.g., quiescent state of cell bodies of neuroblasts or astrocytes) when moving along the blood vessels, so neuroblasts migrate by close adhesion to the vessels through the anterior process. The above studies demonstrate that blood vessels play a key role in the migration of neuroblasts.

## Regulatory Mechanisms of Vasophilic Migration

### Role of Brain-Derived Neurotrophic Factor (BDNF) in Neuroblast Migration with Blood Vessels

RMS, a migratory route that originates in the SVZ of the brain, migrates to reach the OB, where neuroblasts migrate vertically along the scaffold provided by microvascular endothelial cells (MEC) [47]. Within this microvascular network, humeral signals required for the survival and differentiation of neurons become the components of the vascular niche. BDNF secreted from endothelial cells can induce neuroblasts to migrate to the neighboring microvascular territory, working as a neutrophilic factor to maintain neuroblast survival [53]. BDNF fosters neuronal migration via p75NTR (low-affinity BDNF receptor), and neuroblasts in migration can express not only p75NTR but also GABA. Astrocytes neighboring blood vessels stretch out processes to wrap around neuroblasts and express TrkB (high-affinity BDNF receptor). After a while, the migrating cell will enter the astrocyte membranes through TrkB induced by GABA to enter the quiescent state. Grade et al. [59] reported that in ischemic brain injury, BDNF secreted by endothelial cells induces neuroblasts to migrate to the neighboring vascular territory and acts synergistically with astrocytes to promote the migration around the ischemic focus. Wu et al. [1] reported that neuroblasts migrated along the activated astrocytic tunnel, directed by BDNF gradient between subventricular zone (SVZ) and injured cortex after traumatic brain injury. To sum up, the vascular migration of neuroblasts in the adult brain has been found to have a link with the interaction among endothelial cells, astrocytes, and neuroblasts.

### Role of Stromal Cell-Derived Factor 1 α(SDF-1α) in Neuroblast Migration with Blood Vessels

Factors that guide and support the vascular migration pathway of neurons also include endothelial and pericyte-derived cytokines, such as stromal cell-derived factor 1α (SDF-1α) in neuroblast migration. CXCR4, the receptor for SDF-1α, is expressed in neurons of developing and mature brain. SDF-1α and CXCR4 are key factors in the neuroblasts migration [60, 61]. Kokovay et al. [62] discovered highly expressed SDF-1α in the capillary beds within the lateral ventricles, which induces CXCR4-expressing neuroblasts to leave the ependymal environment, and upregulate the integrin. Previous experimental results [44, 63] showed that vascular migration and chain migration of neuroblasts in the brain following stroke are dependent on β1 integrin [64]. The daughter cells generated by the transient proliferation of NSCs can express the laminin receptor α6β1 integrin. Shen Q et al. [65] found that NSCs in the SVZ anchor the vascular basement membrane throughα6β1 integrin. If the cell adhesion and signal transduction mobilized by α6β1 are inhibited, the diffusion of NSCs can be facilitated, suggesting that the interactions between NSCs and laminin can inhibit the diffusion of NSCs in the vascular territory and synergistically regulate the migration of neuroblasts along ventricular mircrovasculature. Zhang et al. [66] in their experiment showed that SDF-1α/CXCR4 signaling in the embryonic brain can regulate the vascular migration of oligodendrocyte precursor cells (OPCs).

### Role of Vascular Endothelial Growth Factor (VEGF) in Neuroblast Migration with Blood Vessels

Vascular endothelial growth factor (VEGF) can directly provide neuroprotection and nutrient supply to nerve cells and glial cells. Both VEGF and VEGFR2 can be expressed in NSC. Administration of VEGF helped establish a vascular niche, reduce infraction rate, and improve neurological recovery after stroke [67]. Moreover, studies on VEGF-overexpressing transgenic mice confirmed neurogenesis is increased in the SVZ, suggesting VEGF could increase the proliferation and survival of the neuroblasts via its VEGFR2 [68]. Furthermore, in vitro experiment demonstrated that VEGF is capable of enhancing neuronal survival as well as inducing axonal growth [69]. Another experiment found VEGF can induce neuroblast migration by releasing chemoattractant through signal pathways [70]. Carmen et al. [71] proved the deposits of matrix-binding VEGF isoforms could guide accurate granule cell migration. All these findings certified the pivotal role of VEGF in neuroblast migration, demonstrating that blood vessels within development stages also regulate the migration through VEGF secretion [45, 72]

### Role of Angiopoietin-1 (Ang-1) in Neuroblast Migration with Blood Vessels

Angiopoietin-1(Ang1), secreted from vascular endothelial cells, acted as chemoattractant for neuroblasts, induced neuroblasts to migrate toward the injured area through the cognitive receptors Tie2 [35, 73]. Ang1 expression was observed within blood vessels extending from the infarct core to SVZ along the pathway of neuroblast migration and adjacent to cells positive for Tie2 [35]. Lin et al. [74] confirmed that following stroke induction, Ang-1/Tie2 are distinct in time and distribution. Ang-1 plays a critical role in the late stage of angiogenesis, as well as vascular remodeling and maturation; while Tie2 is distributed almost exclusively in endothelial cells and is essential for vascular remodeling. According to Beck et al. [75], the upregulation of Ang-1/Tie2 following ischemia allows for the maturation of neovasculature, thus ensuring the maintenance of functional cerebral vasculature. As reported in some studies [76], Ang-1 mRNA and Tie2 expression increased several hours into stroke, and peaked on the 3rd post-stroke day, which lasted for 7 days. Moreover, Lin et al. [74] revealed that Ang-1 mRNA was transiently expressed after stroke and highly expressed at 1–2 weeks.

### Role of Extracellular Matrix (ECM) Proteins in Neuroblast Migration with Blood Vessels

In addition to the above diffusible factors, molecules regulating neuroblasts migration with blood vessels were recently revealed [47, 64]. Vascular basement membrane contains many extracellular matrix (ECM) proteins, such as type 4 collagen, laminin, and fibronectin, which are produced by endothelial cells, vessel-enwrapping pericytes, and astrocytes [47]. The integrins are transmembrane receptors that mediate cell adhesion to the ECM, which is involved in the migration of various cell types. In the adult V-SVZ, NSCs and their progenies including neuroblasts expressβ1 integrins, which bind to multiple ECM proteins.β1 integrin is required for the vasculature-guided migration of neuroblasts toward a lesion in the post-stroke brain [64]. The laminin-integrin-dependent adhesion of neuroblasts to a scaffolding substrate facilitates their migration in vitro [64].

Taken together, after brain injury, chemoattractive/trophic factors such as BDNF, SDF-1, VEGF and Ang-1, and ECM, these factors triggered the vasculature-guided migration of neuroblasts toward the injury (Table 1): (1) Astrocytes modulate the local concentration of BDNF by capturing it with the high-affinity receptor TrkB, while neuroblasts express a low-affinity BDNF receptor p75NTR. This mechanism regulated vasculature-guided neuroblasts migration. (2) SDF-1 and Ang1, secreted from vascular endothelial cells, acted as chemoattractant for neuroblasts, induced neuroblasts to migrate toward the injured area through the cognitive receptors CXCR4 and Tie2, respectively. In other words, SDF1/CXCR4 and Ang-1/Tie2 signaling regulated the neuroblasts migration along blood vessels. (3) VEGF / VEGFR signaling also assisted the vascular migration of neuroblasts. (4) The interaction of β1 integrin expressed in neuroblasts and laminin, an ECM protein composing the vascular basal lamina, facilitates neuroblast migration using vascular scaffolds. However, the role of other factors in the vasculature-guided neuroblasts migration needs to be further explored.

**Table 1:** Brief review of the neuroblasts migration with blood vessels under different brain injury conditions in the literature

Literature (authors and year)	Type of brain injury	Neuroblasts state	Secreted from vascular endothelial cells	Secreted from neuroblast	Secreted from astrocytes
Snapyan, M., et al. 2009; Grade S., et al. 2013	Ischemic brain injury	Migration state	BDNF	p75NTR	-
Grade S., et al. 2013	Ischemic brain injury	Quiescent state	BDNF	GABA	TrkB
Wang, R.Y.et al. 2022 Ma, S., et al. 2021 Tsai, H et al. 2016 Kokovay, E., et al. 2010	Ischemic brain injury Traumatic brain injury The developing brain	Migration state	SDF1	CXCR4	-
Wang, Y., et al. 2007 Ruiz de Almodovar, C., et al. 2010	Ischemic brain injury	Migration state	VEGF	VEGFR2	-
Teng-Nan Lin et al. 2000 Beck, H., et al. 2000 Teng-Nan Lin et al. 2001	Ischemic brain injury	Migration state	Ang-1	Tie2	-
Fujioka, T.et al. 2017	Stroke brain injury	Migration state	ECM	β1 integrin	-

*GABA*, gamma-aminobutyric acid; *BDNF*, brain-derived neurotropic factor; *SDF-1*, stromal cell-derived factor 1; *CXCR4*, C-X-C chemokine receptor type 4; *VEGF*, vascular endothelial growth factor; *VEGFR2*, vascular endothelial growth factor receptor 2; *Ang-1*, angiopoietin-1; *ECM*, extracellular matrix

### Neuroblast Migration in TBI and Other Types of Brain Injury

After TBI or other types of brain injury, some of the neuroblasts in the SVZ migrate toward the site of injury to repopulate the injured tissues [1, 26, 77]. The notable migratory capacity of SVZ-derived neuroblasts is essential for efficient neuronal regeneration in remote areas of the brain. As these neuroblasts migrate for long distances through brain tissues, they are supported by various guidance cues BDNF [1, 17, 26, 53, 59, 78, 79], SDF-1α [17, 26, 60, 61, 78, 80], VEGF [17, 71, 78], and Ang-1 [17, 73, 78], as chemoattractants.

Lee et al. [12] described that the neural progenitors in ischemic striatum were significantly increased on day 5 and 7 post-subarachnoid hemorrhage. Grade et al. [59] reported that many neural progenitors migrated from the SVZ into ischemic area at 2 weeks after ischemic stroke. In aspiration lesion model, neuroblast migration started 2 days post-lesion, and this migration appeared to be persistent even 2 months after lesion [77]. Wu et al. [1] found that neuroblasts migration initiated as early as day 1 and finally arrived at injured cortex on day 7 after TBI in a controlled cortical impact (CCI) model. Apparent discrepancy in these previous studies might arise from differences in lesion models. The specific mechanism needs to be further explored.

## Discussion

Neuroblasts are mainly distributed in the area close to the V-SVZ [17], suggesting their limited ability to reach the injury site. Besides, only portion of the neuroblasts survive and differentiate into mature neurons. In a rodent stroke model, only about 0.2% of the dead neurons were replaced by these new neurons. Thus, the number of new neurons in affected areas that are remote from the V-SVZ (the lateral striatum and neocortex) should be increased to induce an efficient recovery from neurological dysfunction in various pathologies. In experimental animals, the infusion of growth and neurotrophic factors and paracrine signaling molecules successfully enhanced the number of V-SVZ neuroblasts and new neurons in the injured brain.

Most studies have aimed for proliferation and survival of neuroblasts in clinical applications, while a few have focused on promoting neuroblast migration toward the injury site [17]. Efficiency of growth or neurotrophic factors is enhanced if they are administered with biocompatible hydrogels. Artificial scaffolding can also enhance neuronal migration to an injury. Ajioka et al. demonstrated that in neonatal mice, a laminin-rich porous sponge transplanted into the injured cortex functions as a migration scaffold for V-SVZ–derived neuroblasts, leading to the increased number of neuroblasts that reach the lesion [81]. In adult mice after stroke, injectable hydrogels enriched with laminin induced efficient migration of neuroblasts from the V-SVZ toward the striatum [64]. Microfiber or nanofiber biomaterials also improved the migration of neuroblasts from the V-SVZ [17]. Artificial scaffolds support not only migration, but also the survival and differentiation processes of the new neurons, which consequently may promote endogenous neuronal regeneration.

## Conclusion and Outlook

**Fig. 7:**
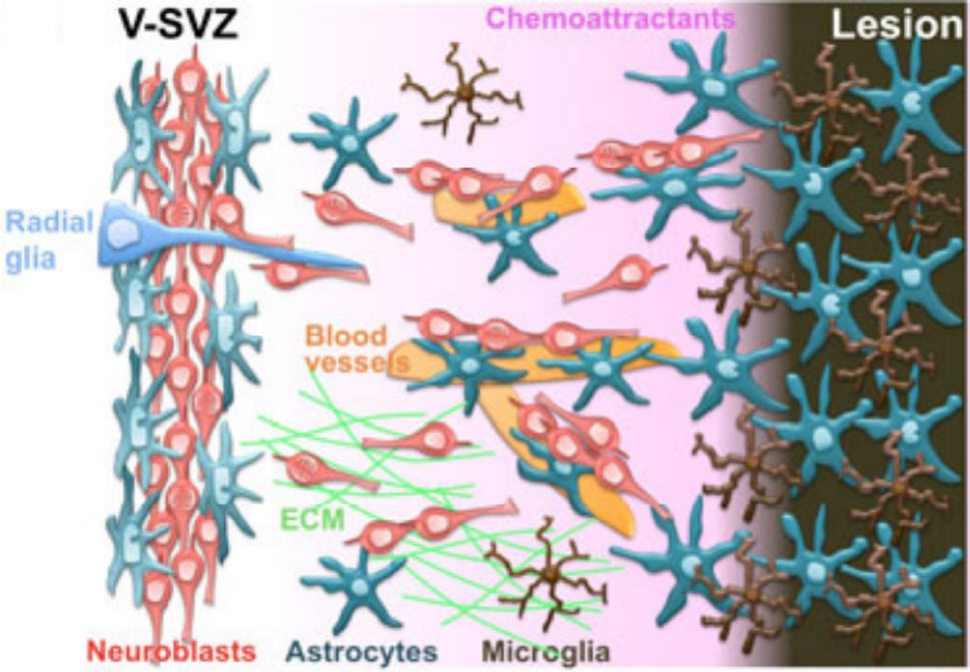
Mechanisms supporting neuroblast migration toward an injury site in the brain [17]. After a brain insult, neuroblasts in the V-SVZ are redirected to the lesion by several diffusible attractive factors secreted by injury-activated astrocytes (blue-green), microglia (brown), and vascular endothelial cells (orange). Migrating neuroblasts use blood vessels, astrocytic processes, radial glial processes (light blue), and extracellular matrices (ECM, yellow-green) as scaffolds

In summary, neuroblast migration presents three modes according to the characteristics of cells that act as scaffolds during the migration process: gliophilic migration, neurophilic migration, and vasophilic migration (Fig. 7). Many signaling molecules, including BDNF, SDF-1, VEGF, Ang-1, and ECM proteins, affect vascular migration, synergistically regulating the migration of neuroblasts to target areas along blood vessels. However, the precise role of blood vessels in the migration of neuroblasts needs to be further explored. Based on the scaffolding function of blood vessels for cell migration, some experiments have confirmed that artificial scaffolding can promote the migration of nerve cells, thereby restoring nerve function after brain injury [46, 82–86]. The in-depth study of neuroblast migration will most probably provide theoretical basis and breakthrough for the clinical treatment of brain injury diseases.

## Data Availability

All data generated during this study are included in this article.
